# Supplementary material for: Assessment of Physical Activity Using Waist-Worn Accelerometers in Hospitalized Heart Failure Patients and Its Relationship with Kansas City Cardiomyopathy Questionnaire
Source: J Clin Med. 2021 Sep 11;10(18):4103. doi: 10.3390/jcm10184103 (PMC8470222; doi:10.3390/jcm10184103)
Supplement: Supplementary file 1 [file jcm-10-04103-s001.zip › jcm-1339911-supplementary.pdf]

Journal of
*Clinical Medicine*

# Assessment of Physical Activity Using Waist-Worn Accelerometers in Hospitalized Heart Failure Patients and Its Relationship with Kansas City Cardiomyopathy Questionnaire

**Yasuyuki Shiraishi [1,*], Nozomi Niimi [1], Ayumi Goda [2], Makoto Takei [3], Takehiro Kimura [1], Takashi Kohno [2], Masataka Kawana [4], Keiichi Fukuda [1] and Shun Kohsaka [1]**

[1] Department of Cardiology, Keio University School of Medicine, Tokyo 160-8582, Japan; figarofuga@gmail.com (N.N.); kimura@z7.keio.jp (T.K.); kfukuda@a2.keio.jp (K.F.); sk@keio.jp (S.K.)
[2] Department of Cardiovascular Medicine, Kyorin University Faculty of Medicine, Tokyo 181-8611, Japan; ayumix34@yahoo.co.jp (A.G.); kohno.ta@gmail.com (T.K.)
[3] Department of Cardiology, Tokyo Saiseikai Central Hospital, Tokyo 108-0073, Japan; makoto_tk@hotmail.com
[4] Department of Medicine, Division of Cardiovascular Medicine, Stanford University, Stanford, CA 94305, USA; mkawana@stanford.edu
* Correspondence: yasshiraishi@keio.jp; Tel.:+81-3-3353-1211; Fax: +81-3-5843-6167

# Table of Contents

**Figure S1**. A summary of the Kansas City Cardiomyopathy Questionnaire scores

**Table S1**. Patient backgrounds in patients undergoing right heart catheterization

**Table S2**. Subgroup analysis on correlation of accelerometer-measured physical activity with the Kansas City Cardiomyopathy Questionnaire for patients receiving right heart catheterization

**Table S3**. Subgroup analysis on correlation of physical activity assessed by cardiopulmonary exercise testing with the Kansas City Cardiomyopathy Questionnaire and accelerometer-measured parameters for patients receiving right heart catheterization

**Figure S1.** A summary of the Kansas City Cardiomyopathy Questionnaire scores.

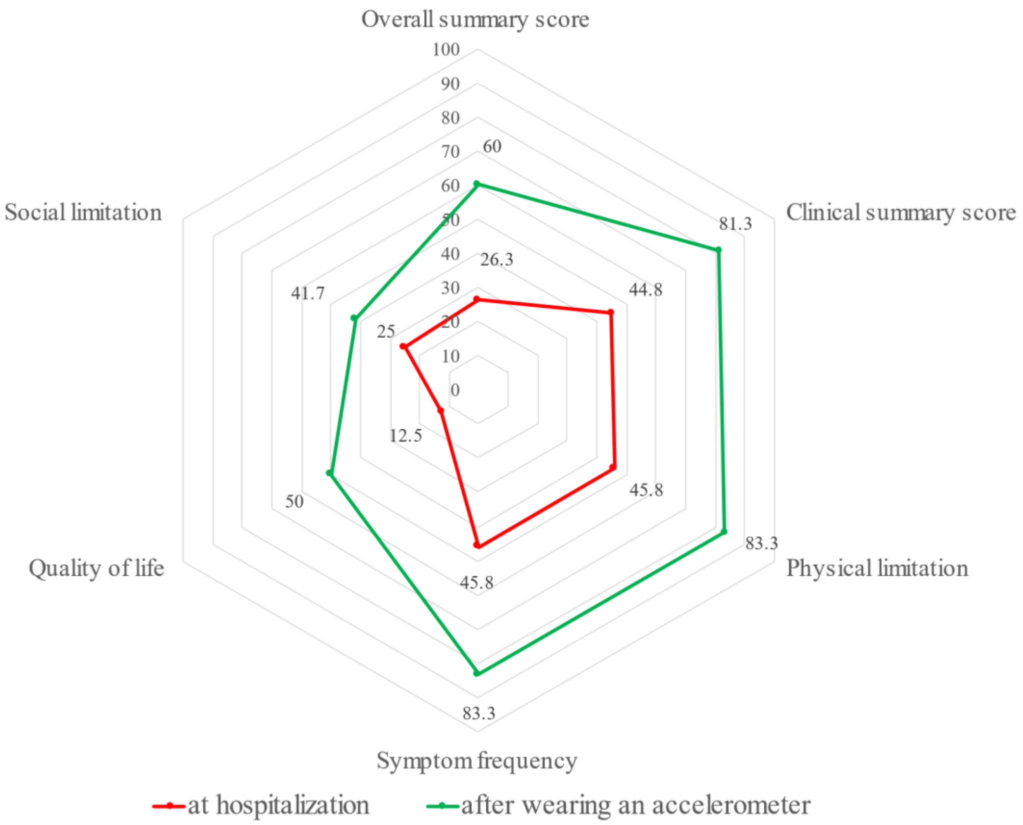

**Table S1.** Patient backgrounds in patients undergoing right heart catheterization.

| Value | Patients with RHC n = 14 |
|---|---|
| Age, years | 69 (55–79) |
| Men, n (%) | 9 (64) |
| Body mass index, kg/m² | 21.0 (20.4–25.5) |
| Systolic blood pressure, mm Hg | 98 (91–107) |
| Heart rate, bpm | 70 (59–74) |
| LVEF, % | 27 (25–34) |
| LVDd, mm | 58 (56–69) |
| LVDs, mm | 51 (44–60) |
| NYHA class, n (%) | |
| II | 10 (71) |
| III | 4 (29) |
| Clinical frailty scale, n (%) | |
| 2 (fit) | 6 (43) |
| 3 (managing well) | 5 (36) |
| 4 (very mild frailty) | 3 (21) |
| 5 (mild frailty) | 0 (0) |
| Triggers of acute decompensation | |
| Nonadherence to diet, n (%) | 0 (0) |
| Nonadherence to medication, n (%) | 1 (7) |
| Overwork, n (%) | 2 (14) |
| Arrhythmia, n (%) | 1 (7) |
| Ischemia, n (%) | 1 (7) |
| Infection, n (%) | 2 (14) |
| Others or none, n (%) | 7 (50) |
| Past medical history | |
| Patients with ≥1 HF hospitalization in previous 1 year, n (%) | 5 (36) |
| Coronary artery disease, n (%) | 3 (21) |
| Atrial fibrillation, n (%) | 3 (21) |
| Hypertension, n (%) | 6 (43) |
| Diabetes mellitus, n (%) | 1 (7) |
| Stroke, n (%) | 5 (36) |
| COPD, n (%) | 2 (14) |
| Laboratory test | |
| Hemoglobin, g/dL | 12.0 (11.1–13.8) |
| Creatinine, mg/L | 1.0 (0.9–1.4) |
| Blood urea nitrogen, mg/L | 23.5 (16.3–32.8) |
| Sodium, mEq/L | 139.1 (137.7–140.3) |
| Potassium, mEq/L | 4.4 (4.1–4.5) |
| Total bilirubin, mg/L | 0.9 (0.7–1.2) |
| Albumin, mg/L | 4.0 (3.5–4.0) |
| BNP at hospitalization, pg/mL | 916 (256–1896) |
| BNP at discharge, pg/mL | 342 (178–432) |
| BNP improvement during hospitalization, % * | 58.4 (47.2–85.2) |
| HF treatment | |
| Loop diuretics, n (%) | 11 (79) |
| ACEi or ARB, n (%) | 13 (93) |

| | |
|---|---|
| **ARNi, n (%)** | 0 (0) |
| **Beta blocker, n (%)** | 13 (93) |
| **MRA, n (%)** | 9 (64) |
| **SGLT2i, n (%)** | 3 (21) |
| **ICD, n (%)** | 2 (14) |
| **CRT, n (%)** | 1 (7) |
| **CPX parameter #** | |
| **Peak $VO_2$, ml/kg/min** | 11.3 (9.7–15.7) |
| **AT $VO_2$, ml/kg/min** | 9.3 (8.6–11.5) |
| **VE vs. $VCO_2$ slope** | 37.1 (30.6–47.9) |
| **Accelerometer-measured parameter** | |
| **Daily step counts, n/day** | 2539 (1263–4252) |
| **Exercise time** | 2.2 (1.1–3.5) |
| **%sedentary time, %** | 71.9 (63.7–82.0) |

* BNP improvement was defined as (BNP at admission – BNP at discharge) / BNP at admission.

Abbreviations: LVEF, left ventricular ejection fraction; LVDd, left ventricular end-diastolic diameter; LVDs, left ventricular end-systolic diameter; NYHA, New York Heart Association; HF, heart failure; COPD, chronic obstructive pulmonary disease; BNP, B-type natriuretic peptide; ACEi, angiotensin-converting enzyme inhibitor; ARB, angiotensin receptor antagonist; ARNI, angiotensin receptor neprilysin inhibitor; MRA, mineralocorticoid receptor antagonist; SGLT2i, sodium glucose cotransporter-2 inhibitor; ICD, implantable cardioverter defibrillator; CRT, cardiac resynchronization therapy; CPX, cardiopulmonary exercise testing; $VO_2$, oxygen consumption; AT, anaerobic threshold; VE, expiratory minute volume; $VCO_2$, carbon dioxide production;.

**Table S2.** Subgroup analysis on correlation of accelerometer-measured physical activity with the Kansas City Cardiomyopathy Questionnaire for patients receiving right heart catheterization.

| Value | Accelerometer-measured parameters | | |
|---|---|---|---|
| | **Daily step count** | **Exercise time** | **%sedentary time** |
| KCCQ-OSS | r = 0.43, P = 0.125 | r = 0.56, P = 0.037 | r = -0.63, P = 0.017 |
| KCCQ-CSS | r = 0.58, P = 0.029 | r = 0.61, P = 0.022 | r = -0.77, P = 0.001 |
| Each domain of KCCQ-12 | | | |
| Physical limitation | r = 0.56, P = 0.037 | r = 0.52, P = 0.056 | r = -0.66, P = 0.011 |
| Symptom frequency | r = 0.52, P = 0.057 | r = 0.60, P = 0.024 | r = -0.77, P = 0.001 |
| Quality of life | r = -0.03, P = 0.931 | r = 0.28, P = 0.337 | r = -0.44, P = 0.118 |
| Social limitation | r = 0.21, P = 0.482 | r = 0.32, P = 0.258 | r = -0.13, P = 0.658 |

r represents Pearson's correlation coefficient. %sedentary time is defined as sedentary time / device-equipped time.

Abbreviations: KCCQ, Kansas City Cardiomyopathy Questionnaire; OSS, overall summary score; CSS, clinical summary score.

**Table S3.** Subgroup analysis on correlation of physical activity assessed by cardiopulmonary exercise testing with the Kansas City Cardiomyopathy Questionnaire and accelerometer-measured parameters for patients receiving right heart catheterization.

| | Cardiopulmonary exercise testing parameters | | |
|---|---|---|---|
| **Value** | **Peak $VO_2$** | **AT $VO_2$** | **VE vs. $VCO_2$ slope** |
| KCCQ-OSS | r = 0.51, P = 0.094 | r = 0.38, P = 0.227 | r = -0.21, P = 0.519 |
| KCCQ-CSS | r = 0.54, P = 0.072 | r = 0.48, P = 0.117 | r = -0.29, P = 0.369 |
| Each domain of KCCQ-12 | | | |
| Physical limitation | r = 0.59, P = 0.046 | r = 0.56, P = 0.057 | r = -0.31, P = 0.328 |
| Symptom frequency | r = 0.44, P = 0.158 | r = 0.34, P = 0.275 | r = -0.23, P = 0.466 |
| Quality of life | r = 0.56, P = 0.061 | r = 0.35, P = 0.260 | r = -0.03, P = 0.920 |
| Social limitation | r = 0.12, P = 0.704 | r = 0.01, P = 0.996 | r = -0.06, P = 0.843 |
| Accelerometer-measured parameters | | | |
| Daily step count | r = 0.15, P = 0.651 | r = 0.25, P = 0.425 | r = -0.27, P = 0.394 |
| Exercise time | r = 0.13, P = 0.682 | r = -0.16, P = 0.616 | r = -0.23, P = 0.476 |
| %sedentary time | r = -0.74, P = 0.006 | r = -0.63, P = 0.028 | r = 0.39, P = 0.211 |

r represents Pearson's correlation coefficient. %sedentary time is defined as sedentary time / device-equipped time.

Abbreviations: KCCQ, Kansas City Cardiomyopathy Questionnaire; OSS, overall summary score; CSS, clinical summary score; $VO_2$, oxygen consumption; AT, anaerobic threshold; VE, expiratory minute volume; $VCO_2$, carbon dioxide production.
